# Does surgery affect systemic immune response? a perioperative analysis of TGF-β, IL-8 and CD45RO

**DOI:** 10.3389/fonc.2023.1307956

**Published:** 2023-12-07

**Authors:** Leah Trumet, Jutta Ries, Niclas Ivenz, Philip Sobl, Falk Wehrhan, Rainer Lutz, Marco Kesting, Manuel Weber

**Affiliations:** ^1^Department of Oral and Cranio-Maxillofacial Surgery, Friedrich-Alexander-Universität Erlangen-Nürnberg (FAU), Erlangen, Germany; ^2^Deutsches Zentrum Immuntherapie (DZI) and Comprehensive Cancer Center Erlangen-EMN (CCC ER-EMN), Friedrich-Alexander-Universität Erlangen-Nürnberg (FAU), Erlangen, Germany; ^3^Department of Operative Dentistry and Periodontology, Friedrich-Alexander Universität Erlangen-Nürnberg (FAU), Erlangen, Germany; ^4^Private Office for Maxillofacial Surgery, Freiberg, Germany

**Keywords:** perioperative systemic changes of signaling pathways immune tolerance, OSCC, HNSCC, surgery, interleukins, oral surgery, cytokines

## Abstract

**Background:**

The options of (neo-)adjuvant immunotherapy in addition to surgery in the treatment of oral squamous cell carcinoma (OSCC) are steadily increasing, but patients do not always respond to therapy as intended. The objectives of this study were to investigate the systemic perioperative course of the biomarkers CD45RO, TGF-β, and IL-8 in non-tumor-related minor and tumor-related major maxillofacial surgery and to perform association analyses with demographic and histomorphologic parameters. A deeper understanding of surgery-related changes in various of different immune biomarkers could help to better understand the immunologic consequences of surgery which could influence immunotherapeutic protocols.

**Methods:**

Peripheral whole blood from 38 patients was analyzed by real-time quantitative polymerase chain reaction (RT-qPCR) at five different timepoints before and after maxillofacial surgery to detect changes in mRNA expression of the biomarkers TGF-β, IL-8 and CD45RO. All patients underwent general anesthesia to undergo either resection and free flap reconstruction for OSCC or minor maxillofacial surgery (controls). Statistical analysis was done using Mann-Whitney-U test, Wilcoxon test, and Spearman’s correlation.

**Results:**

Compared to the preoperative expression, there was a significant postoperative downregulation of CD45RO, TGF-β and IL-8 until the 4th postoperative day (p ≤ 0.003) in OSCC patients. For TGF-β and IL-8, the reduction in expression was significant (p ≤ 0.004) compared to controls. By postoperative day 10, all analyzed parameters converged to baseline levels. Only CD45RO still showed a significant downregulation (p=0.024). Spearman analysis revealed a significant correlation between increased duration of surgery and perioperative reduction in peripheral blood expression of CD45RO, TGF-β and IL-8 (p ≤ 0.004). Perioperative changes in TGF-β and PD-L1 expression were shown to be not correlated. Preoperative TGF-β expression was significantly lower in patients with lymph node metastases (p=0.014).

**Conclusion:**

With regard to the analyzed parameters, major oncologic head-and-neck surgery does not seem to have long-lasting systemic immunologic effects. Reduced CD45RO might be an expression of transient systemic immunosuppression in response to major surgery. The association of duration of surgery with expression changes of immunologic markers supports efforts to keep the duration of surgery as short as possible. As perioperative TGF-β and PD-L1 expression changes are not associated, these results support further investigation of a combined perioperative anti-PD-1 and anti-TGF-β immunotherapy.

## Introduction

Oral squamous cell carcinoma (OSCC) is the most common type of head-and-neck squamous cell carcinoma (HNSCC) (1). The current treatment protocol includes surgery, radiotherapy, chemotherapy, and combined therapies (1). However, despite multimodal therapy, the survival rate of OSCC is low (1).

Immune checkpoint inhibitors (ICIs) have expanded the therapeutic options for OSCC (2, 3). Despite the increasing use of ICIs, approximately 80% of patients with advanced OSCC do not respond to anti-PD1 treatment monotherapy (4–6). Therefore, it is of particular interest to identify possible reasons for non-response (4–6).

The relevance of combination immunotherapy is demonstrated by studies investigating dual targeting of anti-PD-L1 and TGF-β, which could potentially lead to better anti-tumor responses (7). Interestingly, increased activity of the TGF-β pathway has been found in non-responders to anti-PD-(L)1 treatment (7). In a preclinical study, there was evidence of restored sensitivity to anti-PD-L1 therapy by inhibiting of TGF-β (7).

Another approach to improve response rates is to delay the timing of ICI-therapy prior to tumor surgery (5). To optimize this approach, the impact of surgical trauma on systemic immunologic parameters that may influence ICI-therapy needs to be better understood. Therefore, the analysis of perioperative expression changes immune parameters interacting with ICI therapy is of potential relevance. The influence of surgical trauma on PD-L1, FOXP3, IL-6, and IL-10 has already been shown in a perioperative time course (8). Patients who underwent OSCC surgery were found to have higher expression of PD-L1 and IL-10 and lower expression of FOXP3 and IL-6 for a few days after surgery (8). Longer duration of surgery correlated with an increase in the expression of PD-L1 and IL-10, whereas the expression of FOXP3 and IL-6 decreased with increasing duration of surgery, indicating that the duration of surgery should be as short as reasonably possible (8).

Macrophages activation plays a key role in the pathogenesis of several diseases (9). The polarization of macrophages into M1 (pro-inflammatory) and M2 (anti-inflammatory) is influenced by interferon (IFN)-γ or lipopolysaccharides (M1) or by Th2 cytokines such as IL-10 (M2) (9). M2 macrophages contribute to wound healing and tissue repair, but are also tumor promoting (9). M2 shifting is associated with IL-8 production, and high IL-8 expression is associated with downregulated antigen presentation (10, 11).

Under physiological conditions, IL-8 is produced by epithelial cells, monocytes and endothelial cells and its receptors are CXCR1 or CXCR2 (4). Pro-inflammatory IL-8 is a potential indicator of poor outcome in patients with advanced melanoma treated with immune checkpoint inhibitors and it is associated with higher tumor grading and staging (4, 11, 12). IL-8 is considered a potential biomarker for OSCC, as salivary expression of this cytokine is higher in OSCC compared to healthy controls (13).

Currently, therapeutics that inhibit IL-8 or IL-8R (CXCR2) are in late preclinical or clinical development (12). Safety and efficacy studies of combined anti-IL-8 and nivolumab therapy are ongoing (12). Combined anti-CXCR2 and anti-PD1 therapy is expected to be a more effective therapeutic option compared to ICI therapy alone (11).

Neutrophils polarize towards N1 (pro-inflammatory) or N2 (anti-inflammatory) depending on the (tumor) environment and surrounding stimuli. Especially at high concentrations of TGF-β and IL-8, this shift is towards N2 (4). In cancer patients, systemic TGF-β levels are often elevated compared to healthy controls, and high levels of TGF-β are associated with poor prognosis, metastasis, and more aggressive disease in several malignancies (7).

CD45 is a leukocyte antigen that has several cell type and activation/differentiation dependent isoforms (14). The CD45RO isoform is a marker for human memory T cells and is often increased in solid tumors (14). A high density of CD45RO+ T cells in OSCC and colorectal cancer patients is associated with a good clinical outcome. Therefore, an analysis of perioperative expression changes in peripheral blood - which is currently lacking - may be beneficial for therapeutic targeting (2, 15).

The aim of this prospective study was to analyze the mRNA expression of IL-8, TGF-β and CD45RO at different time points in the peripheral blood of patients undergoing complex OSCC tumor resection and reconstruction surgery compared to minor oral surgery procedures. In addition, a correlation analysis was performed between the expression of the aforementioned biomarkers and duration of surgery. Finally, a correlation analysis of TGF-beta expression- changes with previously published PD-L1 and FOXP3 expression changes was performed.

## Materials and methods

### Patients cohort

For this prospective study, a patient population consisting of oral squamous cell carcinoma (OSCC) patients undergoing receiving selective supraomohyoid neck dissection (ND) with microvascular flap reconstruction and patients undergoing minor oral surgery procedures under general anesthesia (controls) was evaluated. All participants were evaluated and treated at the Department of Oral- and Cranio-Maxillofacial Surgery, Universitätsklinikum Erlangen-Nürnberg, Friedrich-Alexander-Universität Erlangen-Nürnberg (FAU). Enrollment in this study, from 2019 to 2021, was performed with the full consent of the patients and controls and with the approval of the local ethics committee (application number 415_20 B).

### Sampling of peripheral blood

The sampling and analysis procedure was performed as previously described in greater detail (8). Whole peripheral venous blood samples were collected the first surgical incision (pre), after the last surgical suture (post), on postoperative day two (2d post), and on postoperative day four (4d post). The 2.5 ml of whole peripheral venous blood per sample was collected in a PAXgene^®^ Blood miRNA Tube (PreAnalytiX GmbH, Hombrechtikon, Switzerland) and inverted 8-10 times, incubated at room temperature for 2 hours, and frozen at -20°C for 24 hours before further processing. This processing was performed at the biological laboratory of the Department of Oral- and Cranio-Maxillofacial Surgery, Universitätsklinikum Erlangen-Nürnberg, Friedrich-Alexander-Universität Erlangen-Nürnberg (FAU).

### Analysis of IL-8, CD450_RO and TFG-β expression by quantitative-real-time-reverse-transcriptase-polymerase-chain-reaction

The RNA concentration was determined using a NanoDrop 1000 spectrophotometer (Thermo Fisher Scientific Company, Waltham, Massachusetts, USA) at wavelength of 260 nm. Transcription of RNA into cDNA was performed using the transcriptor high-capacity kit (Applied Biosystems, Waltham, USA) according to the manufacturer’s specifications.

All samples were centrifuged prior to RT-qPCR in the QuantStudio 6 Pro Real-Time PCR System (Applied Biosystems, Waltham, USA). Primer for IL-8, CD450RO and TFG-β and Power SYBR™ Green PCR Master Mix (Thermo Fisher Scientific, Waltham, USA) were used for specific analysis of the biomarkers. The detailed specifications of the primer are shown in **Table 1**. GAPDH was used as a housekeeping control gene to normalize the CT values (**Table 1**).

**Table 1:** Primer for RT-qPCR analysis.

Real-time qPCR					
Primer	Sequence (5’ to 3’)	Primer length [bp]	Amplicon length [bp]	Annealing temperature [°C]	Accession Number
**CD45RO/s**	TGC CTA CCT TAA TGC CTC TGA A	20	103	60	NM_002838.5/ NM_080921.3 NM_002838.5/ NM_080921.3
**CD45RO/as**	ATC ACA TGT TGG CTT AGA TGG AGT	24	103	60	
**IL-8/s**	CTC TTG GCA GCC TTC CTG AT	20	115	60	NM_001354840.3 NM_000584.4
**IL-8/as**	TGG GGT GGA AAG GTT TGG AG	20		60	
**TGF- β/s**	CAG TGG TTG AGC CGT GGA G	19		60	NM_000660.7
**TGF- β/as**	AGT GAA CCC GTT GAT GTC CA	20	105	60	
**GAPDH/s**	GAC CCC TTC ATT GAC CTC AAC TA	23	122	60	NM_002046.5
**GAPDH/as**	GAA TTT GCC ATG GGT GGA AT	20	122	60	

The table shows the primers that were selected for RT-qPCR mRNA expression analysis of CD45RO, TGF-β and IL-8. Sequence, Primer length, amplicon length, annealing temperature, accession number are also given for each primer. GAPDH served as internal control.

### Statistical analysis

We used SPSS 23 statistical software package (SPSS Inc., Chicago, Illinois, USA) for statistical analysis. We analyzed the ΔCt data from RT-qPCR and calculated the fold change (FC) based on it. After the exploratory data analysis, the Mann-Whitney U test was used to determine significant differences in expression level between the two groups. The Wilcoxon test was used to examine statistical differences in the postoperative course of marker expression at different time points. The ΔΔCt ((ΔΔCt = ΔCt(post) - ΔCt(pre))) method was used to evaluate expression changes in response to the duration of surgery. Spearman’s correlation test was used for correlation analysis.

For between-group analysis, we evaluated the expression differences between each biomarker at different time points. In addition, associations of marker expression with demographic and histomorphologic parameters (T-status, N-status, grading) were tested. Furthermore, we tested correlations with previously published PD-L1 and FOXP3 expression (8) and surgery duration using the Spearman’s correlation test.

A p-value ≤0.05 was considered statistically significant and a p-value ≤0.001 was considered highly significant. A ρ-value (Spearman’s correlation coefficient) ≤0.5 was considered strongly correlated, a ρ-value ≤0.3 was considered moderately correlated, and a ρ-value ≤0.1 was considered weakly correlated.

## Results

The ΔCt_pre_ value was determined by RT-qPCR and used as a baseline value to define the extent of changes in the expression levels (FC) and their significance (p-value) at four different time points. Increased ΔCt values represent decreased mRNA expression. Standard deviation (SD) and ρ-value (Spearman’s correlation coefficient) are shown.

### Patients cohort

The detailed information about the patient population can be found in **Table 2**. Peripheral whole blood samples were collected from patients aged between 30 and 80 years of age. In the OSCC group (n=25), 5 were female and 20 males with an overall mean age of 62 (± 9.53) years old. According to TNM classification, 11 patients had T1/T2, and 14 patients had T3/T4 tumors. 12 out of 25 patients had no lymph node metastasis (N0) and 13 patients had lymph node metastasis (N+).

**Table 2:** Demographic of OSCC and controls.

		OSCC		Controls	
		n	% of cases	n	% of cases
**Number of cases**	**38**	25	65.79	13	34.21
**Gender**	**Male** **Female**	20 5	52.63 13.16	7 6	18.42 15.79
**Mean age ± SD**		62.16 ± 9.53		54.00 ± 19.30	
**Range of age**		41-80		30-80	
		**n**	**% of OSCC cases**		
**T-Status**	**T1** **T2** **T3** **T4**	6 5 6 8	24 20 24 32		
**N-Status**	**N0** **N+**	12 13	48 52		
**Grading**	**G1** **G2** **G3**	0 14 11	0 56 44		

Demographic characteristics of the patient cohort consisting of Controls and OSCC patients.

For both OSCC and the Control group, the number of cases, sex, and age of all patients are given. For the OSCC group, the parameters for T-status, N-status and grading are additionally shown.

Of the 13 patients in the control group, 46% (n=6) were female and 54% (n=7) were male with ana mean age of 54(± 19.30) years. The reasons for general anesthesia and surgery in the controls were minor procedures such as dentialveolar surgery, removal of osteosynthesis material, or similar indications. All patients in the control group were healthy regarding malignant diseases.

### CD45RO

**Figure 1:**
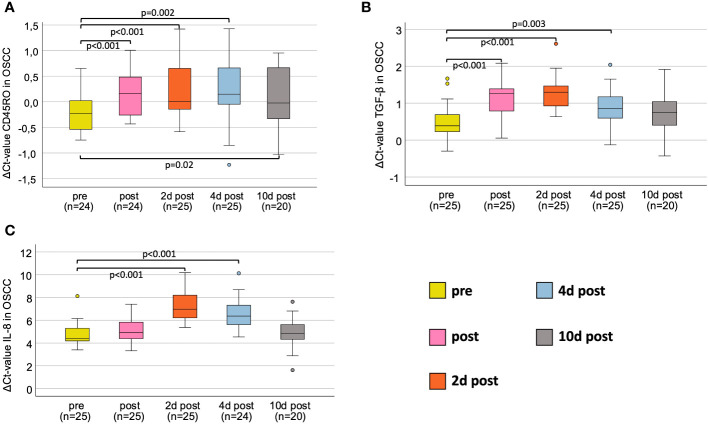
Perioperative expression courses for OSCC-patients for all markers investigated via RT-qPCR. **(A)** Perioperative CD45RO mRNA expression. **(B)** Perioperative TGF-β mRNA expression. **(C)** Perioperative IL-8 mRNA expression. Boxplots showing the median mRNA expression rates of CD45RO **(A)**, TGF-β **(B)** and IL-8 **(C)** in peripheral whole blood samples at five different timepoints (tp) of patients undergoing OSCC tumor resection. The tp were pre-, post-, 2d post-, 4d post- and 10d post-tumor-resection. The different tp are marked in five different colors, described on the right lower part of the figure. Analysis was done using the Wilcoxon-test. n: number of cases. ΔCt value is scaled on the y-axis. p: significant p-values are given.

In the OSCC-group, the progression of CD45RO initially showed a significant decrease in expression immediately after surgery (median ΔCt_post_ =0.16, p<0.001), also on day 2 after surgery (median ΔCt_2dpost_<0.01, p<0.001) and 4^th^ postoperative day (median ΔCt_4dpost_=0.15, p=0.002), followed by an increasing expression approaching baseline 10 days after the surgery (medianΔCt_10post_=0.007, p=0.024) (**Table 3** and **Figure 1A**).

**Table 3:** Parameters of perioperative time courses of CD45RO in OSCC vs. controls.

CD45RO in OSCC vs. Controls	pre	post	2d post	4d post
CD45RO in OSCC				
sample size (n)	24	24	25	25
median ΔCt-value	-0.23	0.16	<0.01	0.15
average ( ± standard derivation)	-0.23 ( ± 0.37)	0.15 ( ± 0.42)	0.23 ( ± 0.56)	0.24 ( ± 0.62)
CD45RO in Controls				
sample size (n)	13	13	6	4
median ΔCt-value	-0.04	-0.13	-0.02	0.12
average ( ± standard derivation)	-0.12 ( ± 0.36)	-0.10 ( ± 0.38)	-0.12 ( ± 0.34)	0.11 ( ± 0.08)
**fold-change**	1.08	0.84	0.79	0.91
**p-value**	0.28	0.11	0.31	0.88

The table shows the analyzed sample size (n), average ( ± SD), median ΔCt value, fold-change (FC) and p-value to “pre” of CD45RO-expression. CD45RO peripheral blood mRNA expression was analyzed in OSCC and Controls. Those data are demonstrated for 4 different time points (pre, post, 2d post, 4d post). FC was determined using the ΔΔCt method, which compared the average ΔCt values of the different time points, starting from the preoperative ΔCt value.

The significant p-values are written in bold letters.

OSCC: Primary tumor resection, microvascular free flap reconstruction and selective neck dissection due to oral squamous cell carcinoma.

Controls: Minor maxillofacial surgeries.

**Figure 2:**
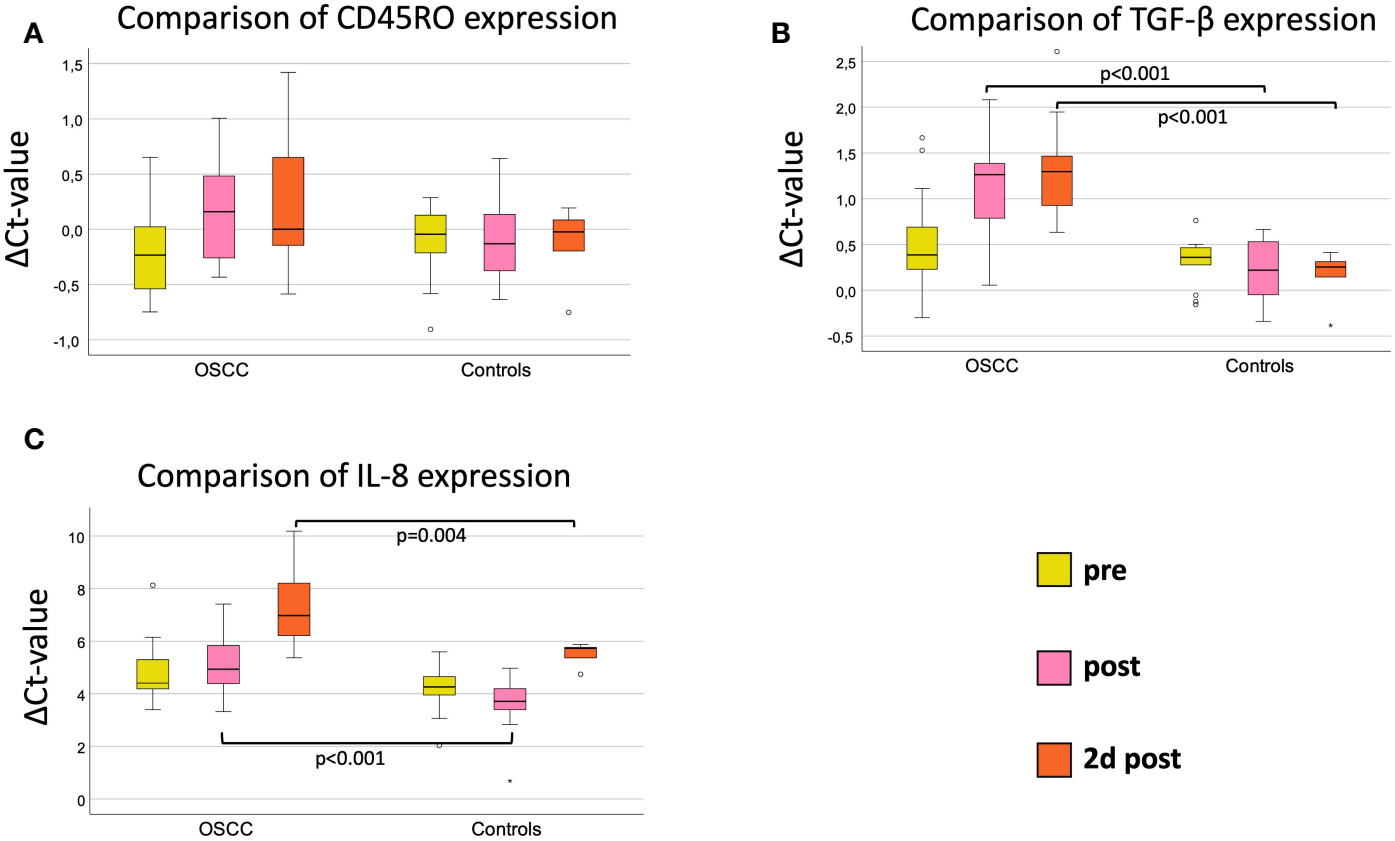
Comparison of marker expression on three perioperative time points (pre, post and 2d post) between OSCC and controls. **(A)** Perioperative CD45RO mRNA expression. **(B)** Perioperative TGF-β mRNA expression. **(C)** Perioperative IL-8 mRNA expression. Boxplots showing the median mRNA expression rates of CD45RO **(A)**, TGF-β **(B)** and IL-8 **(C)** in peripheral whole blood samples at 3 different timepoints (tp) of OSCC-patients versus Controls. The tp were pre-, post- and 2d post-surgery. The different tp are marked in three different colors, described on the right lower part of the figure. Mann-Whitney-U test. n: number of cases. ΔCt value is scaled on the y-axis. p: significant p-values are given.

Comparing both groups, there were no significant expression changes in CD45RO expression during the postoperative course (**Figure 2A**). The median ΔCt_pre_ value was -0.23 in OSCC and -0.04 in controls (p=0.28, FC=1.08) (**Table 3** and **Figure 2A**). The highest fold-change (1.15) between OSCC and controls was reached preoperatively (p=0.28) (**Table 3**).

**Figure 3:**
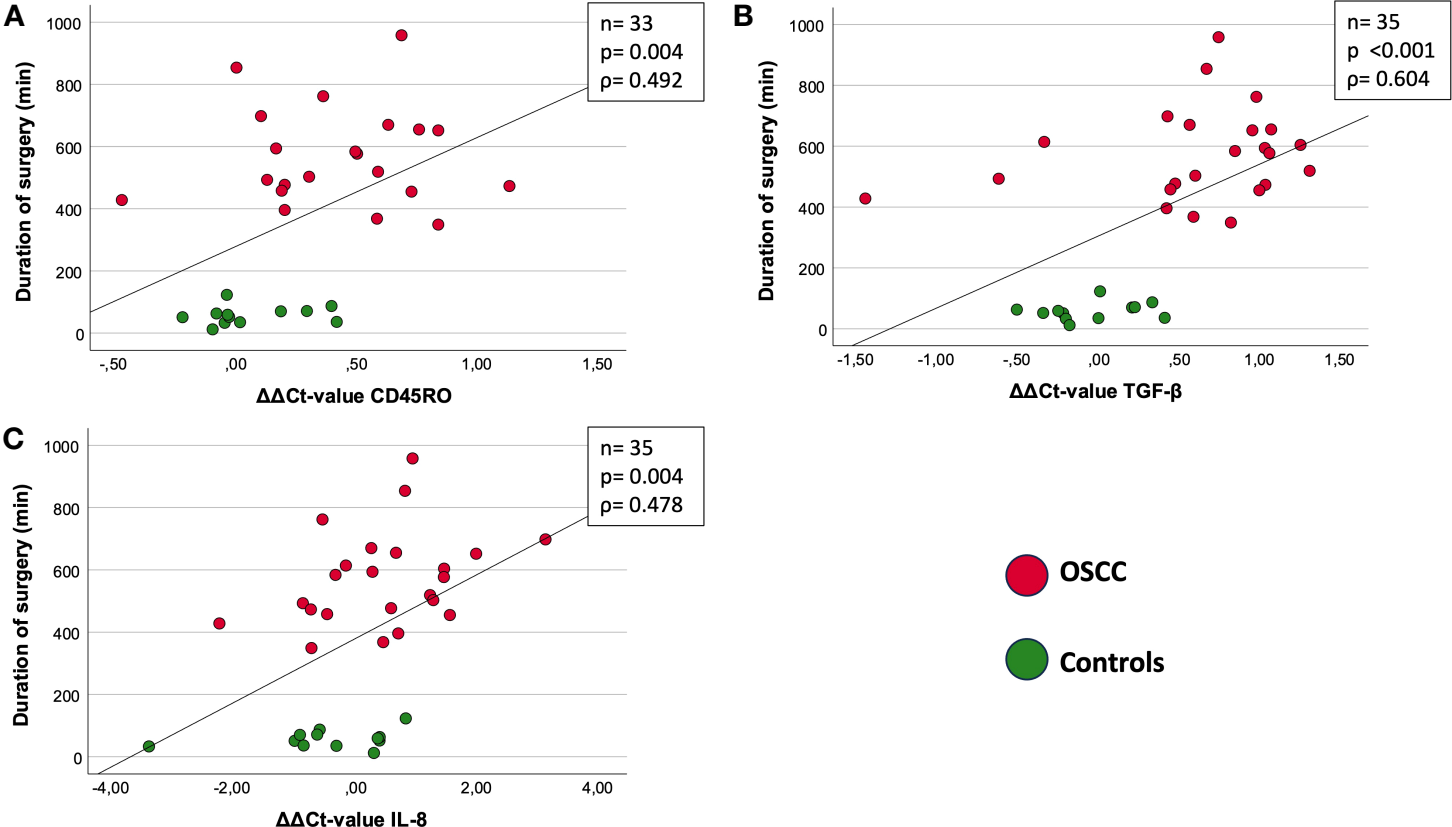
Correlation between duration of surgery [min] and ΔΔCt value (ΔΔCt = ΔCt(post) – ΔCt(pre)). **(A)** CD45RO: ρ = 0.492; p=0.004. **(B)** TGF-β: ρ =0.604; p<0.001. **(C)** IL-8: ρ= 0.478; p=0.004. The figures show the correlation between marker expression of CD45RO **(A)**, TGF-β **(B)** and IL-8 **(C)** and duration of surgery. Both OSCC and Controls are included in the present correlation. The different groups are shown and explained by the two different colors (red and green). The measure of linear correlation was made doing the Spearman correlation test. The duration of surgery was measured in minutes (min) and the expression was measured using the ΔΔCt value, which was calculated by subtracting the ΔCt_post_ from ΔCt_pre_-value. ρ: Spearmans-correlation coefficient. ΔΔCt value (ΔΔCt = ΔCt(post) – ΔCt(pre)). n: number of cases. p: significant p-values are given.

In both groups, the duration of surgery was correlated with the ΔΔCt-value ((ΔΔCt = ΔCt(post) – ΔCt(pre))) of CD45RO (n=33) (**Figure 3A**). There was a highly significant correlation of increasing duration of surgery with decreasing expression of CD45RO (p=0.004, ρ=0.492) (**Figure 3A**).

### TGF-β

In OSCC, the course of TGF-β initially showed a significant decrease in expression immediately after surgery (ΔCt_post_=1.26, p<0.001), peaking at 2 daysafter surgery (ΔCt_2dpost_=1.3, p<0.001), followed by an increasing expression (ΔCt_4dpost_=0.86, p=0.003), approaching baseline at 10 days after surgery (ΔCt_10post_=0.86; p=0.073) (**Table 4** and **Figure 1B**).

**Table 4:** Parameters of perioperative time courses of TGF- β in OSCC vs. controls.

TGF-β in OSCC vs. Controls	pre	post	2d post	4d post
TGF-β in OSCC				
sample size (n)	25	25	25	25
median ΔCt-value	0.39	1.26	1.30	0.86
average ( ± standard derivation)	0.51 ( ± 0.46)	1.09 ( ± 0.52)	1.27 ( ± 0.45)	0.88 ( ± 0.50)
TGF-β in Controls				
sample size (n)	13	13	6	4
median ΔCt-value	0.36	0.22	0.25	0.36
average ( ± standard derivation)	0.31 ( ± 0.27)	0.23 ( ± 0.10)	0.17 ( ± 0.28)	0.37 ( ± 0.25)
**fold-change**	1.15	1.82	2.14	1.43
**p-value**	0.44	**<0.001**	**<0.001**	**0.04**

The table shows the analyzed sample size (n), average ( ± SD), median ΔCt value, fold-change (FC) and p-value to “pre” of TGF- β-expression. TGF- β peripheral blood mRNA expression was analyzed in OSCC and Controls. Those data are demonstrated for 4 different time points (pre, post, 2d post, 4d post). FC was determined using the ΔΔCt method, which compared the average ΔCt values of the different time points, starting from the preoperative ΔCt value.

The significant p-values are written in bold letters.

OSCC: Primary tumor resection, microvascular free flap reconstruction and selective neck dissection due to oral squamous cell carcinoma.

Controls: Minor maxillofacial surgeries.

When comparing OSCC and controls, there were highly significant expression changes during the postoperative course (**Figure 2B**). The median ΔCt_pre_ value was 0.39 in OSCC and 0.36 in controls (p=0.44, FC=1.15) (**Table 4** and **Figure 2B**). Immediately after surgery, the ΔCt_post_-value increased to 1.26 in OSCC and decreased to 0.22 in controls, resulting in high expression difference (p<0.001, FC=1.82) (**Table 4** and **Figure 2B**). On postoperative day 2, the median ΔCt_2dpost_ increased to 1.30 in the OSCC group and further decreased to 0.17 in the controls (p<0.001, FC=2.14). In the later postoperative course on day 4, TGF-β expression increased in the OSCC group increased with a ΔCt_4dpost_ of 0.86 in OSCC and ΔCt_4dpost_ of 0.36 in controls (p=0.004, FC=1.43) (**Table 4** and **Figure 2B**).

In both groups, the duration of surgery was correlated with the ΔΔCt-value ((ΔΔCt = ΔCt(post) – ΔCt(pre))) of TGF-β (n=35) (**Figure 3B**). There was a highly significant correlation of increasing duration of surgery with decreasing expression of TGF-β (p<0.001, ρ=0.604) (**Figure 3B**).

### IL-8

In oral cancer patients, the course of IL-8 initially showed highly significant decrease in expression on day 2 postoperatively (ΔCt_2dpost_=6.97, p<0.001) compared to preoperatively (ΔCt_pre_=4.41), followed by an increasing expression on day 4 postoperatively (ΔCt_4dpost_=6.36, p<0.001) approximating baseline 10 days after surgery (ΔCt_10dpost_=4.82, p=0.411) (**Table 5** and **Figure 1C**).

**Table 5:** Parameters of perioperative time courses of IL-8 in OSCC vs. controls.

IL-8 in OSCC vs. Controls	pre	post	2d post	4d post
IL-8 in OSCC				
sample size (n)	25	25	25	24
median ΔCt-value	4.41	4.93	6.97	6.37
average ( ± standard derivation)	4.76 ( ± 1.05)	5.06 ( ± 1.06)	7.25 ( ± 1.40)	6.56 ( ± 1.32)
IL-8 in Controls				
sample size (n)	13	13	6	4
median ΔCt-value	4.26	3.71	5.73	5.39
average ( ± standard derivation)	4.16 ( ± 0.92)	3.66 ( ± 1.10)	5.53 ( ± 0.42)	5.34 ( ± 0.84)
**fold-change**	1.52	2.64	3.29	2.33
**p-value**	0.20	**<0.001**	**0.004**	0.059

The table shows the analyzed sample size (n), average (± SD), median ΔCt value, fold-change (FC) and p-value to “pre” of IL-8-expression. IL-8 peripheral blood mRNA expression was analyzed in OSCC and Controls. Those data are demonstrated for 4 different time points (pre, post, 2d post, 4d post). FC was determined using the ΔΔCt method, which compared the average ΔCt values of the different time points, starting from the preoperative ΔC value.

The significant p-values are written in bold letters.

OSCC: Primary tumor resection, microvascular free flap reconstruction and selective neck dissection due to oral squamous cell carcinoma.

Controls: Minor maxillofacial surgeries.

Comparing IL-8 expression in controls and OSCC, there were significant differences in median ΔCt-values postoperatively (p<0.001, FC=2.64) and on day 2 postoperatively (p=0.004, FC=3.29) with higher IL-8 expression in controls (**Table 5** and **Figure 2C**). On day 4, the median ΔCt value of IL-8 was not significantly different between groups (p=0.059, FC=2.33) (**Table 5**).

In both groups, the duration of surgery was correlated with the ΔΔCt-value ((ΔΔCt = ΔCt(post) – ΔCt(pre))) of IL-8 (**Figure 3C**). There was a highly significant correlation of increasing duration of surgery with decreasing expression of TGF-β (p=0.004, ρ=0.478) (**Figure 3C**).

### TGF-β correlation with PD-L1 and FOXP3

**Figure 4:**
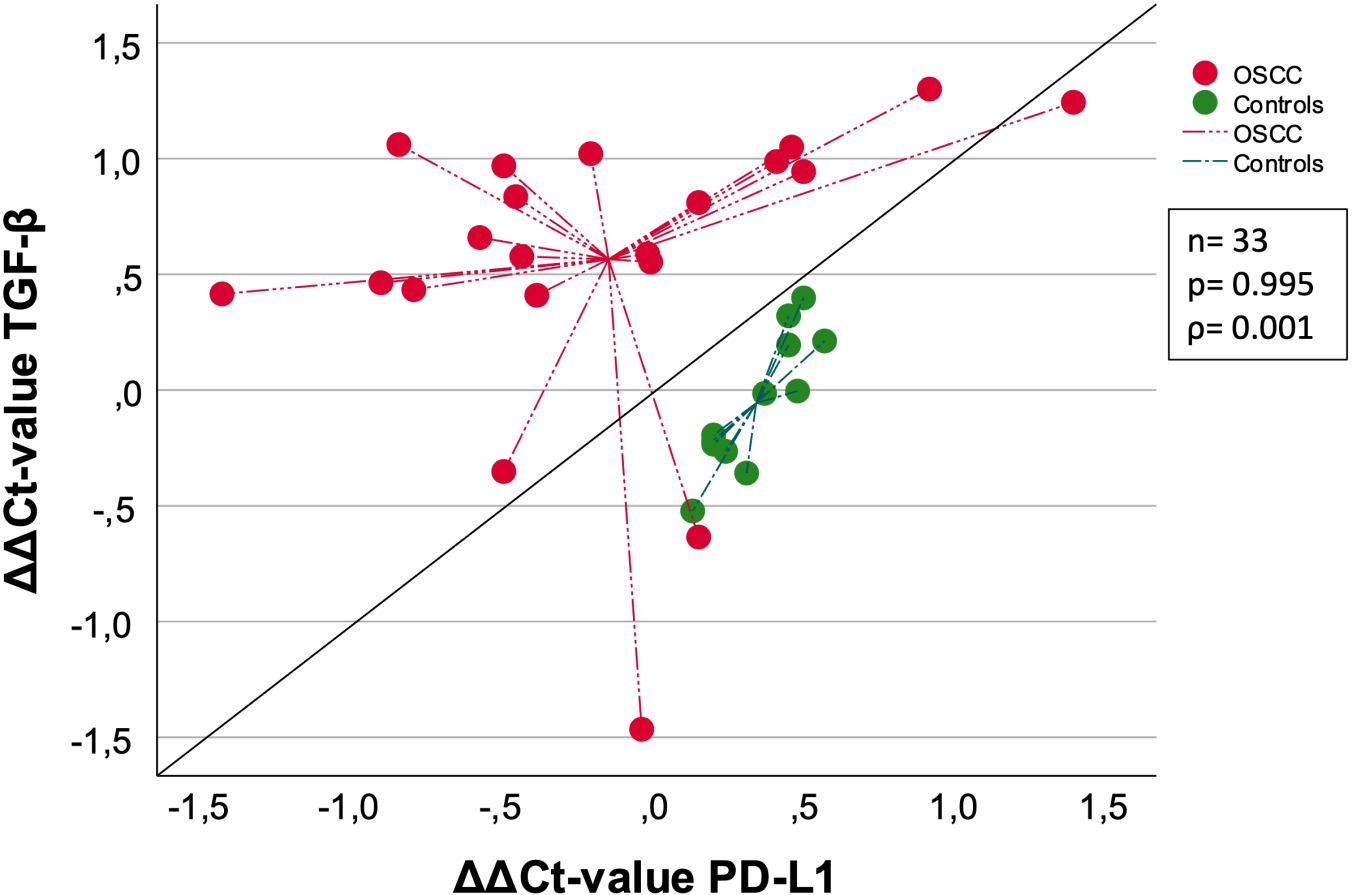
Correlation of OSCC and Controls between TGF-β [ΔΔCt] and PD-L1 [ΔΔCt]. This schematic shows the correlation between TGF-β and PD-L1. Both OSCC (red) and Controls (green) are included in this analysis, separated by the two different colors. The measurement of linear correlation was made doing the Spearman correlation test. The centroid illustration leads to the median ΔΔCt value. The expression of TGF-β and PD-L1 was measured using the ΔΔCt values, which was calculated by subtracting the ΔCt_post_ from ΔCt_pre_-values. The median ΔΔCt value was indicated by the lines connected to centroid for each group. ρ: Spearmans-correlation coefficient. ΔΔCt value (ΔΔCt = ΔCt(post) – ΔCt(pre)). n: number of cases. p: p-value is given.

Correlation analysis of 33 samples between TGF-β and previously published PD-L1 ΔΔCt-values (8) showed a Spearman correlation coefficient of 0.001 and a p-value of 0.995 indicating no correlation (**Figure 4**). The ΔΔCt values are determined calculating ΔCt_post_ – ΔCt_pre_. Controls and OSCC show individual clustering, indicating two separate groups.

In summary, the expression changes of TGF-β are greater compared to PD-L1. The median ΔΔCt value of PD-L1 was higher and the median ΔΔCt value of TGF-β was lower when comparing controls with OSCC (**Figure 4**).

Analysis of expression changes correlating TGF-β and FOXP3 was also performed. Analyzing 33 patients, a Spearman-correlation coefficient of 0.751 and p-value of <0.001 indicated a strong and highly significant positive correlation between TGF-β and FOXP3 expression.

### Demographic and histomorphologic parameters

We analyzed the ΔCt values of all biomarkers pre- and postoperatively between male (n=27) and female (n=11) patients. Only the ΔCt_post_ value of IL-8 and TGF-β reached statistical significance between the sexes, in both cases the biomarker expression was higher in women (p_postIL-8 =_ 0.009, ΔCt_postIL-8-female_=12.18, ΔCt_postIL-8-male_=22.58; p_postTGF-β_ =0.049, ΔCt_postTGF-β-female_=13.91, ΔCt_postTGF-β-male_=21.78). To further characterize the cohort, we also examined the male and female distribution in OSCC (n=25) and Controls (n=13) separately, and only ΔCt_postIL-8_OSCC_ in OSCC showed significance of p=0.006 with again lower ΔCt value of females (ΔCt_postIL-8-female_OSCC_=05.20, ΔCt_postIL-8-male_OSCC_=14.95). There was no significant difference in controls.

**Figure 5:**
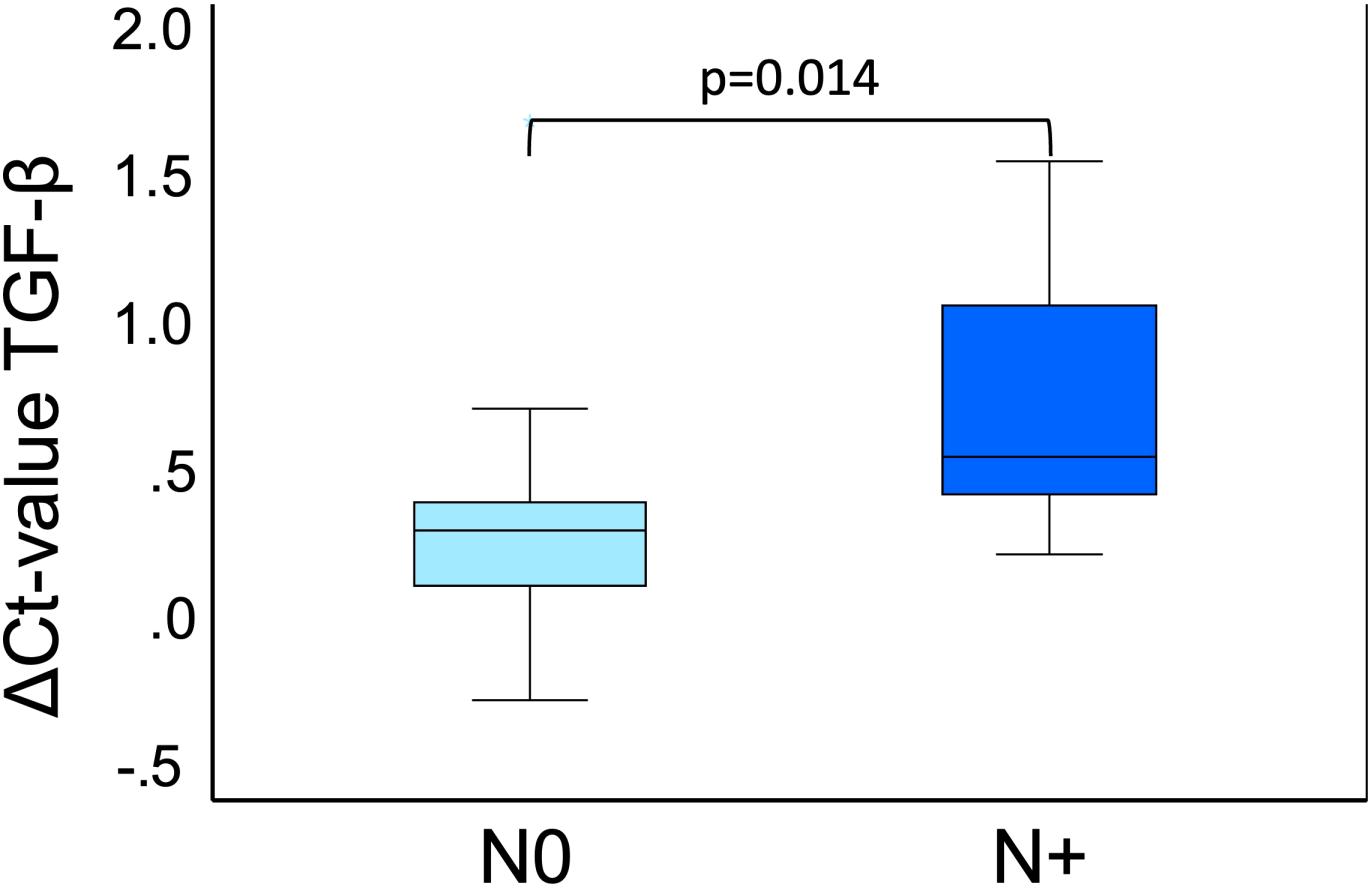
TGF-β expression pre-op in N0 and N+. This boxplot shows the TGF-β expression preoperatively of OSCC 25 cases divided into N0 (n=13) and N+ (n=12). N0 means no lymph node metastases are present, and N+ means lymph node metastases are present. Both N0 (light blue) and N+ (dark blue) are included in this analysis, separated by the two different colors. The significant p-value is given in the boxplots. Mann-Whitney-U test was used for analysis. ΔCt value is scaled on the y-axis. p: significant p-value is given.

In OSCC, we also examined T-status, N-status, and grading of all patients to evaluate whether there were differences in expression between subgroups. Specifications for the subgroups can are shown in **Table 5**. There was no significant difference in the expression of CD45RO and IL-8 with respect to T status, N status and grading. TGF-β expression showed no association with T-status and tumor grading. However, there was a significant expression difference regarding the ΔCt_pre_ value of TGF-β in N0 (n=13) and N+ (n=12) (ΔCt_N0 =_ 0.35, ΔCt_N+_=0.69; p=0.014) (**Figure 5**).

## Discussion

In the current study, we demonstrated that major maxillofacial oncologic surgery including OSCC tumor resection, neck dissection and microvascular reconstruction is associated with a significant postoperative decrease in systemic expression of the cytokines TGF-β and IL-8, as well as the memory cell marker CD45RO. These changes were evident immediately postoperatively until 4 days postoperatively, with no significant difference from the preoperative values at 10 days postoperatively, except for CD45RO. This suggests that the systemic changes we observed in response to major oncologic head and neck surgery are transient. Comparing the OSCC group with controls who received minor surgery, there was a significantly reduced expression of TGF-β and IL-8, but not of CD45RO.

In patients with gastric cancer, reduced serum protein expression levels of TGF-β were associated with more aggressive disease and shorter survival (16). This finding seems counterintuitive at first because TGF-β is an immunosuppressive, anti-inflammatory cytokine that is associated with tumor-promoting cells such as Treg cells (17, 18). In the current study, in addition to reduced TGF-β expression in response to OSCC surgery, we could also find an association between lower preoperative TGF-β mRNA expression levels and lymph node metastases. This suggests a possible association of lower systemic TGF-β mRNA expression with a more aggressive disease. A study in OSCC cell culture showed a direct growth inhibitiory effect of TGF-β on OSCC tumor cells (19). This suggests that the role of TGF-β in OSCC on tumor cells and on immune cells and their bidirectional connection may be relatively complicated and needs further research, especially in the context of potential dual PD-1 and TGF-β inhibition in the future.

As we have previously shown a decrease in FOXP3 expression in OSCC patients in response to surgical therapy (8), and there was also a strong significant positive correlation between decreased TGF-β and FOXP3 expression, these findings support a potential decreased peripheral Treg response in the postoperative period after OSCC surgery, which would be associated with decreased FOXP3 and TGF-β levels.

ELISA analyses showed that there was no significant difference in peripheral blood TGF-β expression in thyroid cancer patients compared to healthy controls (20). A similar result was found in OSCC patients, in whom also no significant difference in TGF-β protein expression could be detected compared to healthy controls before therapy (21). These data are consistent with the similar levels of TGF-β expression at the mRNA level we found in OSCC patients compared to controls before surgery.

In breast cancer patients, surgical tumor resection was associated with a decrease in peripheral blood TGF-β protein expression in the peripheral blood (22). Lack of TGF-β decrease was associated with residual tumor or lymph node metastasis (22). In renal cancer, ELISA analysis revealed a significant decrease in peripheral blood TGF-β levels until the third postoperative day. Thereafter, an increase in TGF-β levels was observed until day 5, which did not reach the preoperative levels (23). These results are analogous to the data of the present study. However, it should be considered that ELISA measurements in the peripheral blood can also detect tumor- derived TGF-β, whereas the m-RNA analyses of the current study can selectively detect the cytokine expression of blood cells and thus represent the response of the peripheral immune system.

Furthermore, we could show that the perioperative expression changes (ΔCt(post) – ΔCt(pre)) of TGF-β are not correlated with expression changes of PD-L1. This suggests that systemic expression changes in response to surgery of both parameters are differentially regulated. This supports concepts of combined blockade of TGF-β and PD1/PD-L1 for perioperative immunotherapy. The weak correlation between PD-L1 and TGF-β expression changes supports findings of non-redundant activities of tumor-evasive mechanisms associated with PD-L1 and TGF-β (7).

In the current study, the expression of IL-8 before surgery was not significantly different between OSCC and tumor-free patients. In the OSCC group, the mean expression of IL-8 was significantly decreased for a period of 4 days after surgery, and in addition, a significant difference in expression between controls and OSCC was examined after surgery.

When comparing the preoperative peripheral blood IL-8 expression of early-stage breast cancer patients with controls, no significant difference was found. In addition, there was no difference in preoperative expression levels at 10 days postoperatively (24). This is analogous to the results of the current study where we found similar IL-8 mRNA levels on day 0 and day 10 with a decrease in IL-8 expression only in the early postoperative period.

An ELISA analysis of IL-8 in the peripheral blood did not show significant differences between OSCC patients and healthy controls (25). In addition, there was no association between clinicopathologic parameters and IL-8 expression (25). Patients who underwent esophageal resection for carcinoma showed a decrease in peripheral blood IL-8 expression by ELISA analysis on days 1, 5 and 7 after surgery compared to the pre-operative value (26). These data are consistent with the results of the current analysis at the mRNA level.

Studies have shown that elevated serum IL-8 expression levels in patients prior to ICI therapy are associated with a less favorable prognosis for ICI efficacy (12). Elevated pre-ICI serum IL-8 levels in patients with melanoma, non–small cell lung cancer, small cell lung cancer and renal cell carcinoma patients are associated with adverse clinical outcomes (12). Furthermore, it has been shown that there is only a weak correlation between immunohistochemically determined PD-L1 expression in tumor samples and baseline serum IL-8 levels in different types of malignancies (12). In a clinical trial of HNSCC patients treated with neoadjuvant anti-PD1 therapy, it was shown that HPV-positive patients who did not respond to ICI therapy had elevated blood levels of IL-8. IL-8 levels decreased in response to ICI therapy, but remained elevated in the non-responder group compared to the ICI responders (27).

Higher serum IL-8 levels in several malignancies prior to ICI therapy with PD-1 or CTLA-4 inhibitors correlate with poorer survival, and this finding was even stronger with single-agent versus combined ICI therapy (4). In addition, data from a pilot study suggest that an increase in serum IL-8 concentration during ICI therapy is an indicator of resistance to this treatment (4). The increased expression of TGF-β and IL-8 in women immediately after surgery-op compared to men suggests that women and men may respond differently to surgical trauma. Due to the small number of cases in the female group, this finding should be further investigated in a larger cohort.

The expression of CD45RO in OSCC changed significantly within 10 days after surgery, reaching a significant decrease in expression at 2 days, 4 days, and 10 days after surgery. However, there was no significant difference in expression compared with the control group that underwent minor surgery. This suggests that the expression changes in CD45RO tend to be less profound than those in TGF-β and IL-8.

In solid tumors, CD45RO positive T-cells are often increased (2). One research group suggested that the very presence of these CD45RO+ T-cells may trigger an immune response to prevent recurrence and metastasis after primary tumor resection (2).

Higher levels of CD45RO+ tumor-infiltrating lymphocytes (TILs) have been shown to be associated with improved overall survival in several cancer types (28). A histologic analysis of CD3, CD4, CD8, and CD45RO in TILs in primary laryngeal squamous cell carcinoma was published in a recent study (28). The most frequent TILs in both the invasive front and the tumor center were CD45RO+, while the least frequent population were CD8+ cells (28). Another study showed that increased intratumoral infiltration of CD45RO cells in HNSCC was associated with prolonged overall- and disease-free survival (29). However, a subgroup analysis of cases with high grading and positive lymph nodes showed an association of high CD45RO with decreased survival (29). These data suggest a complex role of CD45RO in HNSCC.

In breast cancer, an increased infiltration of CD45RO+ cells was shown to be associated with improved survival (30). In colorectal cancer, an increased CD45RO expression in the peripheral blood, as determined by flow cytometry, was associated with smaller tumors, fewer lymph node metastases, and increased overall- and disease-free survival (31). These data suggest that high intratumoral and peripheral CD45RO expression may be beneficial in solid malignancies. Few studies have investigated the role of CD45RO+ T-cells in OSCC (2). The reduction of CD45RO expression in the peripheral blood in response to OSCC surgery demonstrated in the current study suggests a at least transient systemic immunosuppression in a surgically treated patient population.

There was a significant correlation between the decrease in expression (ΔCt(post) – ΔCt(pre)) of CD45RO, TGF-β and IL-8 and the duration of surgery. This finding suggests that the duration of surgery is the critical factor influencing the systemic change in immunologic parameters. These results support efforts to keep the operative time as short as possible in oncologic headandneck surgery (8).

Consistent with our previously published results (8), the data of the current study show that systemic immunologic alterations induced by ablative and microvascular reconstructive OSCC surgery are transient in nature and disappear by 10 days after surgery.

The only perioperative biomarker that still showed a significant difference in expression 10 days after surgery compared to the preoperative value was CD45RO. Based on the approximation to of the expression level to the preoperative value, we assume that the observed decrease in CD45RO expression is a temporary effect, and that the preoperative expression level could be reached in the further time course after 10 days. Thus, to our knowledge, there is no evidence for any long-lasting systemic immunologic alterations with surgical OSCC therapy. These findings are relevant because the combination of surgery and immunotherapy is considered to be a promising approach with the potential to increase OSCC cure rates and improve overall therapeutic outcomes.

## Limitations of the study

This prospective study has some minor limitations. On the one hand, the effect of general anesthesia cannot be studied, and therefore the impact of anesthesia in this regard is unknown. On the other hand, all patients underwent general anesthesia, allowing comparison of the patient cohort.

In addition, the investigation of invasiveness of surgery, the effect of pharmaceuticals and blood transfusions cannot be studied separately. These parameters could also influence systemic immune parameters in addition to the trauma caused by the surgery itself.

However, this situation reflects the clinical routine and thus also the surgical situation as best as possible, since all these parameters can’t be considered in isolation in a clinical cohort.

Another limitation is the 10-day time course, which does not include long-term systemic changes in the immune system. In this regard, the influence of potentially applied adjuvant radio(chemo)therapy should be analyzed in future studies, allowing a deeper understanding of immunomodulatory effects during oral cancer treatment.

There were no deaths directly related to the surgical procedure. We did a follow-up investigation at the beginning of 07/23 to check if the patients were still alive. 4 patients already died and 13 are still alive, the status of the other patients is unknown. To show the reproducibility of this study, a larger number of patients is needed in future research.

## Conclusion

The results of the present study indicate perioperative changes in major primary oncologic head-and-neck surgery with complex reconstruction. However, these changes in the expression of CD45RO, TGF-β and IL-8 are already decreasing within 10 days and therefore indicate an only a transient immunological shift. Therapeutic relevance is mainly shown by the correlation of the expression changes of immunological parameters with increasing duration of surgery, which encourages to keep the duration of surgery as short as possible.

As perioperative TGF-β and PD-L1 expression changes are not associated, these results support combined (neo-)adjuvant anti-PD1 and anti-TGF-β immunotherapy in the context of current clinical trials.

## Data availability statement

The raw data supporting the conclusions of this article will be made available by the authors, without undue reservation.

## Ethics statement

The studies involving humans were approved by Ethikkommission des Universitätsklinikums Erlangen. The studies were conducted in accordance with the local legislation and institutional requirements. The participants provided their written informed consent to participate in this study.

## Author contributions

LT: Formal analysis, Visualization, Writing – original draft, Writing – review & editing. JR: Methodology, Supervision, Writing – review & editing. NI: Data curation, Investigation, Writing – review & editing. PS: Data curation, Investigation, Writing – review & editing. FW: Conceptualization, Writing – review & editing. RL: Validation, Writing – review & editing. MK: Resources, Supervision, Writing – review & editing. MW: Conceptualization, Formal analysis, Investigation, Project administration, Supervision, Writing – review & editing.
